# Unique Features of Germline Variation in Five Egyptian Familial Breast Cancer Families Revealed by Exome Sequencing

**DOI:** 10.1371/journal.pone.0167581

**Published:** 2017-01-11

**Authors:** Yeong C. Kim, Amr S. Soliman, Jian Cui, Mohamed Ramadan, Ahmed Hablas, Mohamed Abouelhoda, Nehal Hussien, Ola Ahmed, Abdel-Rahman Nabawy Zekri, Ibrahim A. Seifeldin, San Ming Wang

**Affiliations:** 1 Department of Genetics, Cell Biology and Anatomy, College of Medicine, University of Nebraska Medical Center, Omaha, Nebraska, United States of America; 2 Department of Epidemiology, College of Public Health, University of Nebraska Medical Center, Omaha, Nebraska, United States of America; 3 Gharbiah Cancer Society, Gharbiah Population-based Registry, Tanta, Egypt; 4 Faculty of Engineering, Cairo University, Giza, Egypt; 5 Egypt National Cancer Institute, Cairo University, Giza, Egypt; Meharry Medical College, UNITED STATES

## Abstract

Genetic predisposition increases the risk of familial breast cancer. Recent studies indicate that genetic predisposition for familial breast cancer can be ethnic-specific. However, current knowledge of genetic predisposition for the disease is predominantly derived from Western populations. Using this existing information as the sole reference to judge the predisposition in non-Western populations is not adequate and can potentially lead to misdiagnosis. Efforts are required to collect genetic predisposition from non-Western populations. The Egyptian population has high genetic variations in reflecting its divergent ethnic origins, and incident rate of familial breast cancer in Egypt is also higher than the rate in many other populations. Using whole exome sequencing, we investigated genetic predisposition in five Egyptian familial breast cancer families. No pathogenic variants in *BRCA1*, *BRCA2* and other classical breast cancer-predisposition genes were present in these five families. Comparison of the genetic variants with those in Caucasian familial breast cancer showed that variants in the Egyptian families were more variable and heterogeneous than the variants in Caucasian families. Multiple damaging variants in genes of different functional categories were identified either in a single family or shared between families. Our study demonstrates that genetic predisposition in Egyptian breast cancer families may differ from those in other disease populations, and supports a comprehensive screening of local disease families to determine the genetic predisposition in Egyptian familial breast cancer.

## Introduction

Familial breast cancer is a hereditary disease and genetic predispositions play major roles in increasing the risk of the disease in the carriers. Genetic predispositions for approximately half of familial breast cancers have been determined, and studies are actively going on to determine the unknown genetic predispositions for the remaining cases [1–3]. Recent studies demonstrate that genetic predispositions for familial breast cancer can be ethnic-specific, as well exemplified by the different spectrum of germline mutation in *BRCA1* and *BRCA2* between different ethnic populations [4–10]. Knowledge of ethnic-specific genetic predispositions for familial breast cancer is important, as it directly affects the accuracy of clinical diagnosis and intervention in patients of different ethnicities. However, current predisposition information is predominantly derived from Western populations. Using the information as the sole reference is not adequate and can potentially lead to misdiagnosis for the patients of non-Western ethnicities, which constitute the majority of human populations.

Egypt population has high-degree of genetic diversity due to its complex and diverse ethnic origins. The population has substantial variations from other populations including its proximal Ethiopia population and distal Yoruba population within African continent [11]. Breast cancer is the most common cancer in Egyptian females with unique characters. While its incidence rate of 45.4 per 100,000 is moderate comparing to other ethnic populations [12], it has high-degree family history of breast cancer, possibly related to high rate of consanguineous marriage in the population [13], and it has high-degree of inflammatory breast cancer [14]. Efforts have been made to study genetic predisposition for Egyptian familial breast cancer, mostly focused on *BRCA1* and *BRCA2* [15], but comprehensive data at genomic level from local patients are lacking.

We used Egyptian familial breast cancer as a model to investigate ethnic-specific genetic predisposition in familial breast cancer. In the study, we applied exome sequencing to analyze genomic variations across all coding genes in five Egyptian breast cancer families. Our study revealed that these disease families have high genetic variability, and they do not contain currently known predispositions for the disease but carry Egyptian-specific genetic variants, some of which may represent Egyptian-specific predispositions. The study supports the concept of ethnic-specific predispositions in familial breast cancer.

## Methods

### Breast cancer families used in the study

The Institutional Review Board of University of Nebraska Medical Center approved the study (049-14-EP). All participants provided verbal informed consent that was read by a study nurse with another nurse or a relative witnessing the delivery of the consent. Written consent was not obtained because of the high illiteracy rate among women in the study population in Egypt. Signatures of the nurse/relative witnessing the interviews were obtained. The local IRB committee in Egypt approved this consent procedure. Five Egyptian breast cancer families from Gharbiah district, Egypt, participated in the study. The families were identified from the Gharbiah Cancer Registry, Egypt. Each participant was interviewed by local oncologists and answered the questions in a standard questionnaire. Venous blood was collected from each participant during the interview process.

### Exome sequencing

DNA was extracted from blood cells using a FlexiGene DNA kit (Qiagen, Valencia, USA). Exome sequences were collected according to previously described procedures [15]. Briefly, genomic DNA was fragmented using a Covaris II system (Covaris, Woburn, MA, USA). Exon templates were isolated using the TruSeq Exome Enrichment Kit (Illumina, San Diego, CA, USA) and exome sequences were collected in a HiSeq2500 sequencer (paired-end 2×150) at 100x coverage. The total variants called from the exome data from this study have been deposited in DRYAD Digital Repository with accession ID: doi:10.5061/dryad.p236p.

### Variant identification

Three controls were used in the study, including 1) the human variation databases of dbSNP, 1000 Genomes and ESP6500 were used to filter out population polymorphism; 2) the Egyptian genome variation data were used to filter out Egyptian-specific normal polymorphism; 3) the variants from 27 Caucasian familial breast cancer probands were used to compare the genomic variation in familial breast cancer families between the two ethnic populations.

Exome sequences were mapped to the human reference genome sequences hg19 [16] using the Burrows-Wheeler Aligner [17] and pre-processed with Picard Toolkit [18]. Variants were called using Freebayes [19], and filtered with a minimum read depth of 10, a minimum of four reads mapped to the location and a minimum of four reads on opposite strands, and a minimum base quality score of 30. Qualified variants were annotated with ANNOVAR [20] against the following reference databases: RefSeq (February 4, 2016), 1000 Genomes (August 2015), NHLBI Exome Sequencing Project (ESP6500) version 2, dbSNP Build 144, and ClinVar (May 5, 2016). Variants causing codon changes were identified, and further filtered by 1000 Genomes with a minor allele frequency (MAF) < = 1%. The remaining variants were further filtered through Egypt population polymorphism data. The Egyptian variant dataset containing 1,422 Egyptian-specific variants was derived from whole genome sequences of 25 Egyptian individuals. Each was sequenced by Ion Torrent technology with base quality score (50+) at average depth of 20X. Variants were called by using the Torrent Suite software following manufacturer’s instruction and the variants present in other ethnic populations at the frequency > 0.01 were eliminated [21]. Annotation was made by using ANNOVAR and in-house programs. Damaging variants were predicted using SIFT [22, score < 0.05] and PolyPhen2 [23, score > 0.909]. Only variants shared by at least two breast cancer-affected members in the same family were included in the final list of damaging variants. Pathways affected by variant-affected genes were identified by searching in the Reactome pathway database (version 57) [24].

## Results

### Breast cancer families used in the study

**Fig 1:**
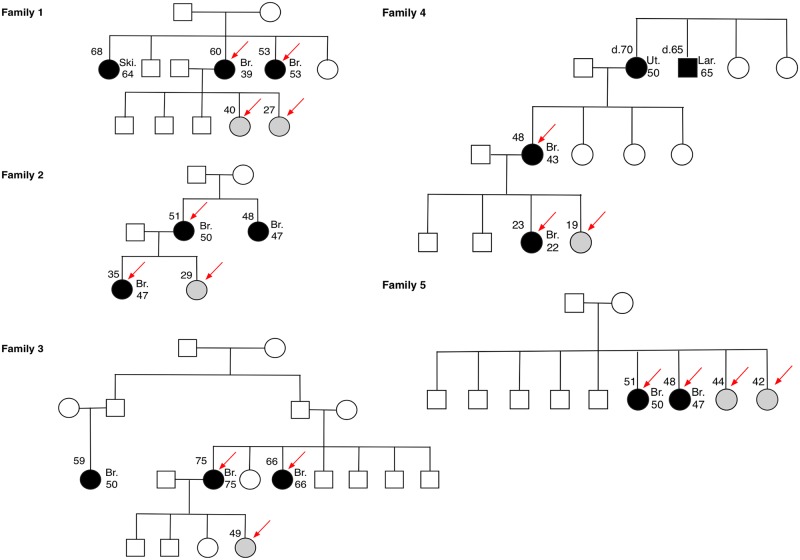
Pedigrees of the five Egyptian familial breast cancer families used in the study. Dark circle: cancer-affected family member; gray circle: cancer-unaffected family member: red arrow: cased used for exome sequencing. Br: breast cancer; Ski: skin cancer; Ut: uterus cancer; Lar: laryngcarcinoma; d: age of death.

We analyzed five Egyptian breast cancer families (Fig 1, Table 1). Familial breast cancer was diagnosed using the inclusion criteria of at least one first-degree relative with breast cancer irrespective of age. In Family 1, three of the four sisters were affected by cancer, of whom two were breast cancer; in Family 2, both sisters and one daughter were affected by breast cancer; in Family 3, two sisters and one cousin were affected by breast cancer; in Family 4, grandmother, grandmother’s brother, mother and a daughter were affected by cancer, of whom mother and daughter had breast cancer; in Family 5, two sisters were affected by breast cancer. Of the 12 breast cancer-affected cases in the five families, eight were diagnosed at an age of younger than or at 50 years old. Based on the availability of DNA samples, 10 breast cancer-affected and seven breast cancer-unaffected family members were included for exome sequencing.

**Table 1:** Clinical data of the breast cancer-affected cases used in exome sequencing.

Case	Diagnosis age	ER	PR	HER2/neu	Lymphnode	Ki-67	Diagnosis
Family 1							
1	39	+	+	-	6/15	5%	Ductual carcinoma, invasive & In situ
2	53	+	+	-	6/19	40%	Ductual carcinoma, invasive & In situ
Family 2							
1	50	+	+	-	0/18	80%	Ductual carcinoma, invasive
2	47	-	-	+	2/24	NA	Ductual carcinoma, invasive & In situ
Family 3							
1	75	+	+	-	0/18	80%	Ductual carcinoma, invasive
2	66	+	+	-	13/14	NA	Ductual carcinoma, invasive & In situ
Family 4							
1	43	+	+	unknown	NA	NA	Ductual carcinoma, invasive
2	22	-	-	-	0/20	5%	Ductual carcinoma, invasive
Family 5							
1	50	+	+	-	1/19	30%	Ductual carcinoma, invasive & In situ
2	47	-	-	-	0/20	5%	Ductual carcinoma, invasive

### Variants in *BRCA1*, *BRCA2* and other known breast cancer predisposition genes

A total of 938,606 unique variants were called from exome sequences of all cases through bioinformatics analysis. To determine if any of these five families carried *BRCA* mutations, we searched the entire variants and identified 18 variants in *BRCA1* and 20 variants in *BRCA2*. Based on Breast Cancer Information Core (BIC) and ClinVar databases, none of the variants was classified as pathogenic (Table 2). We further identified 340 variants in other known predisposition genes of *ATM*, *BARD1*, *BRIP1*, *CDH1*, *CHEK2*, *MRE11A*, *MUTYH*, *NBN*, *NF1*, *PALB2*, *PTEN*, *RAD50*, *RAD51C*, *RAD51D*, *STK11*, and *TP53*. Six variants were identified in *BRIP1*, *MRE11A*, *NBN*, *PTEN*, *TP53*, of which only one in *NBN* (chr8:90990521T>C, NM_002485, c.A511G, p.I171V) was predicted as deleterious by both SIFT and Polyphen2 programs and classified as pathogenic by ClinVar database but this variant was present only in one breast cancer-affected case (member 2 in Family 3). All other variants were predicted as possibly damaging or deleterious by a single program and classified as unknown, untested, non-pathogenic by ClinVar (S1 Table). We also searched the variants affecting 160 cancer-related genes (https://dnapittcrew.upmc.com/db/hsa.php), and identified three coding-change variants affected *RECQL4*, a DNA helicase involved in DNA replication and repair and known to relate with breast cancer [25]. However, the homozygote G-del variant was present in all ten cancer-affected and seven cancer-unaffected cases, the C to T variant was present in an affected and an unaffected members in family 4, and the G to A variant was present in one affected and two unaffected members in family 1. A C to T variant was also identified in *RRM2B*, a gene involved in a TP53-dependent DNA repair process. This variant was present in one affected and two unaffected members in family 5. None of the variants were predicted to damage the function of *RECQL4* and *RRM2B*. Therefore, these variants were unlikely the potential predisposition but the normal variation in these families (S2 Table). The lack of pathogenic variants in *BRCA1*, *BRCA2* and other predisposition genes indicates that these five families are all *BRCAx* breast cancer family [15].

**Table 2:** Variants in *BRCA1* and *BRCA2*.

Position	Exon	Variation type	Variant (HGVS)		Frequency	dbSNP144	Zygocity	Total cases		Classification	
			cDNA	Protein				Affected	Unaffected	BIC	ClinVar
*BRCA1*											
Exonic	11B	nonsynonymous	c.2077G>A	p.Asp693Asn	0.0335	rs4986850	het	1	1	Pending	Benign
Exonic	11B	synonymous	c.2082C>T	p.Ser694=	0.3365	rs1799949	het	7	5	Class 1	Benign
Exonic	11B	synonymous	c.2311T>C	p.Leu771=	0.3353	rs16940	het	7	5	Class 1	Benign
Exonic	11C	nonsynonymous	c.2612C>T	p.Pro871Leu	0.5439	rs799917	het	8	7	Class 1	Benign
Exonic	11C	nonsynonymous	c.3113A>G	p.Glu1038Gly	0.3357	rs16941	het	7	5	Pending	Benign
Exonic	11D	nonsynonymous	c.3548A>G	p.Lys1183Arg	0.3526	rs16942	het	7	5	Pending	Benign
Exonic	13	synonymous	c.4308T>C	p.Ser1436=	0.3363	rs1060915	het	7	5	Class 1	Benign
Exonic	16	nonsynonymous	c.4837A>G	p.Ser1613Gly	0.3558	rs1799966	het	7	5	Pending	Benign
Intronic	6	-	c.213-161A>G		0.5485	rs799912	het	2	3	Class 1	Benign
Intronic	8	-	c.442-34C>T		0.0986	rs799923	het	4	4	Class 1	Benign
Intronic	8	-	c.547+146A>T		0.3526	rs8176140	hom	1	0	Class 1	Benign
Intronic	9	-	c.548-58delT		0.3349	rs273902772	het	7	5	Pending	Benign
Intronic	13	-	c.4357+117G>A		0.0643	rs3737559	het	5	2	Class 1	Benign
Intronic	15	-	c.4485-63A>G		0.3534	rs273900734	het	7	5	Pending	Benign
Intronic	17	-	c.5774+6C>G		.	rs80358032	het	1	0	Pending	Benign / Uncertain
Intronic	17	-	c.4987-68G>A		0.3546	rs8176234	het	7	5	Pending	Benign
Intronic	17	-	c.4987-92A>G		0.3546	rs8176233	het	7	5	Pending	Benign
Intronic	18	-	c.5152+66G>A		0.3425	rs3092994	het	7	5	Class 1	Benign
*BRCA2*											
Exonic	10	nonsynonymous	c.865A>C	p.Asn289His	0.0737	rs766173	het	1	1	Pending	Benign
Exonic	10	nonsynonymous	c.1114A>C	p.His372Asn	0.2494	rs144848	het	7	4	Class 1	Benign
Exonic	10	synonymous	c.1365A>G	p.Ser455=	0.0737	rs1801439	het	1	1	Class 1	Benign
Exonic	11A	synonymous	c.2229T>C	p.His743=	0.0735	rs1801499	het	1	1	Class 1	Benign
Exonic	11B	nonsynonymous	c.2971A>G	p.Asn991Asp	0.0801	rs1799944	het	1	1	Class 1	Benign
Exonic	11B	synonymous	c.3396A>G	p.Lys1132=	0.2668	rs1801406	het	4	3	Class 1	Benign
Exonic	11C	synonymous	c.3807T>C	p.Val1269=	0.1681	rs543304	het	1	2	Class 1	Benign
Exonic	11D	synonymous	c.4563A>G	p.Leu1521=	0.9740	rs206075	hom	10	7	Class 1	Benign
Exonic	11F	synonymous	c.6513C>G	p.Val2171=	0.9736	rs206076	hom	10	7	Pending	Benign
Exonic	14	synonymous	c.7242A>G	p.Ser2414=	0.2326	rs1799955	het	4	3	Class 1	Benign
Exonic	14	nonsynonymous	c.7397C>T	p.Ala2466Val	0.9758	rs169547	hom	10	7	Class 1	Benign
Intronic	2	-	c.67+82C>G		0.0010	rs189026060	het	1	0	Pending	Benign
Intronic	4	-	c.425+67A>C		0.0743	rs11571610	het	1	1	Class 1	Benign
Intronic	5	-	c.426-89T>C		0.0743	rs3783265	het	1	1	Class 1	Benign
Intronic	8	-	c.681+56C>G		0.1859	rs2126042	het	2	0	Pending	Benign
Intronic	14	-	c.7435+53C>T		0.0725	rs11147489	het	1	1	Pending	Benign
Intronic	17	-	c.7806-14T>C		0.5316	rs9534262	hom	9	5	Pending	Benign
Intronic	22	-	c.8755-66T>C		0.5116	rs4942486	hom	7	3	Class 1	Benign
UTR5	2	-	c.-26G>A		0.2093	rs1799943	het	3	3	Class 1	Benign
UTR3	27	-	c.10362A>C		0.1607	rs15869	het	2	2	Class 1	Benign

### Removal of Egyptian-specific normal polymorphism

**Fig 2:**
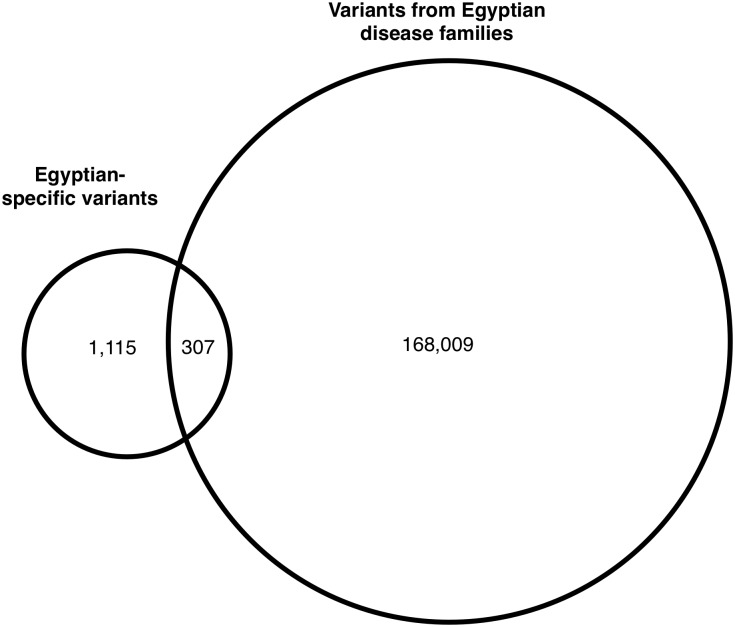
Removal of Egyptian-specific polymorphism. The variants called from exome data and filtered from 1000 Genomes and ESP6500 databases were further filtered through the Egyptian-specific normal variants from Egyptian population. This step eliminated 307 Egyptian-specific normal variants, of which 13 were coding-change variants, from the variants called from the disease families.

The total variants called from the exome sequences were filtered from the normal population polymorphisms from 1000 Genomes and NHLBI Exome Sequencing Project (ESP6500). As the Egypt genomic variation data were not well represented in public databases, the remaining 168,009 variants were further filtered against the 1,422 Egyptian-specific normal variant data derived from Egyptian genome study [21, S3 Table]. This step eliminated 307 Egyptian-specific normal variants, of which 13 were coding-change variants (Fig 2). From the remaining variants, we identified 421 rare, coding-change variants in the five families (S4 Table).

### Comparison of variants between Egypt and Caucasian *BRCAx* familial breast cancer cases

**Fig 3:**
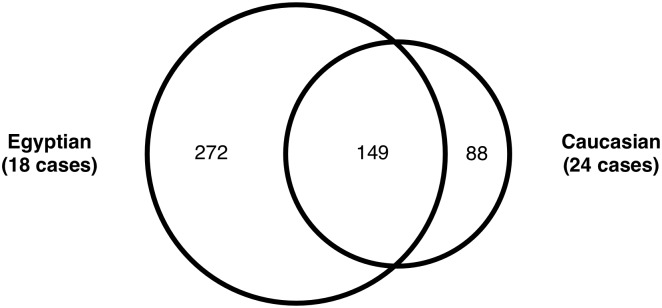
Comparison between coding-damage variants identified in Egyptian and Caucasian familial breast cancer groups. More variants were present in Egyptian group than in Caucasian group, despite the smaller size of Egyptian group than Caucasian group. Nearly two-thirds of Caucasian variants were shared in Egyptian group, but these shared ones accounted for only about a third in Egyptian group.

We compared the 421 coding-change variants with these from 24 Caucasian *BRCAx* cases we identified previously by exome sequencing [17, S5 Table]. Despite the fact that the 18 cases were from five families and the 24 cases were the probands representing 24 families, the number of variants in Egyptian cases (421 variants) was much larger than these in the Caucasian cases (237 variants). There were 149 variants shared between the two groups, but these shared variants accounted for only 35.4% of the total variants in Egyptian group comparing to 62.8% in the Caucasian group. The information indicated that the coding-change variants in Egyptian *BRCAx* familial breast cancer families were more heterogeneous than in the Caucasian *BRCAx* familial breast cancer families (Fig 3).

### Identification of damaging variants in each family

Damaging variants were predicted from the coding-change variants using SIFT and Polyphen2 programs. Those only present in a single case in each family were removed to avoid individual differences, and the remaining ones were present in at least two breast cancer-affected members in each family. The inclusion of unaffected family members aimed to know the status of the damaging variants identified in the cancer-affected members in the family: negative implies they did not carry the potential risk imposed by these damaging variants, positive implies they carried the potential risk considering that they were all at the age of younger than 50 year old. Specific conditions used in each family were:

Family 1: a variant must be shared between both affected sisters, but is not required in either unaffected daughters;
Family 2: a variant must be shared in the affected mother and daughter 1, but is not required in the unaffected daughter 2;
Family 3: a variant must be shared between the two affected sisters, but is not required in the unaffected daughter;
Family 4: a variant must be shared between the affected mother and daughter, but is not required in the unaffected daughter;
Family 5: a variant must be shared between the affected sister 1 and sister 2, but is not required in the unaffected sisters 3 and 4.

A total of 26 distinct damaging variants were identified from the five disease families, of which 19 (73.1%) were known variants in the dbSNP database, 22 (84.6%) were nonsynonymous single nucleotide variants, 25 (96.2%) were heterozygous (Table 3). These variants were distributed at the frequencies of 4 to 9 per family. None of these variants was listed in the ClinVar database (Tables 3 and 4).

**Table 3:** Summary of damaging variants and the affected gene.

Item	Number (%)
Total unique variants	26 (100)
Known	19 (73.1)
Novel	7 (26.9)
Nonsynonyous SNV	22 (84.6)
Frameshift deletion	3 (11.5)
Frameshift substitution	1 (3.8)
Heterozygote	24 (92.3)
Homozygote	2 (7.7)
Gene affected	23

**Table 4:** Damaging mutations identified in five Egyptian familial breast cancer families*.

Gene	Affected	Member				Chr.	Position	Ref.	Variant	Genotype	Var type	Coding change	dbSNP144	Damage		Frequency			ClinVar
														SIFT	PolyPhen2	All	African	Egypt	
Family 1	No.	1	2	3	4														
	Status	A.	A.	U.	U.														
ABCA10	2	+	+	-	+	17	67145196	AG	-	Het.	Frameshift deletion	c.4510_4511del:p.L1504fs	-	-	-	-	-	-	-
CHST15	2	+	+	-	-	10	125780753	GG	-	Hom.	Frameshift deletion	c.1365_1366del:p.P455fs	rs746518074	-	-	-	-	-	-
GRIP1	2	+	+	+	+	12	66935707	C	T	Het.	Nonsynonymous SNV	c.G160A:p.V54I	rs199768740	-	D	0.0004	-	-	-
LOC100129697	2	+	+	-	-	16	89016677	G	C	Het.	Nonsynonymous SNV	c.G151C:p.V51L	rs71395347	D	-	-	-	-	-
LOC100129697	2	+	+	-	-	16	89017569	G	C	Het.	Nonsynonymous SNV	c.G1043C:p.G348A	rs34847212	D	-	-	-	-	-
LOC100129697	2	+	+	+	-	16	89017602	C	T	Het.	Nonsynonymous SNV	c.C1076T:p.P359L	rs188085712	D	-	-	-	-	-
LOC388813	2	+	+	+	-	21	15974379	A	C	Het.	Nonsynonymous SNV	c.T280G:p.Y94D	rs370454293	D	-	0.0020	0.0008	-	-
NBPF10	2	+	+	+	-	1	145296478	G	T	Het.	Nonsynonymous SNV	c.G400T:p.D134Y	rs6663523	D	-	-	-	-	-
PABPC3	2	+	+	-	-	13	25671271	AAGC	GAT	Het.	Frameshift substitution	c.935_938GAT	-	-	-	-	-	-	-
Family 2	No.	1	2	3															
	Status	A.	A.	U.															
C16orf62	2	+	+	-		16	19566981	C	G	Het.	Nonsynonymous SNV	c.C197G:p.A66G	rs564734737	D	-	0.0002	-	-	-
KRTAP21-3	2	+	+	-		21	32090989	C	T	Het.	Nonsynonymous SNV	c.G89A:p.C30Y	rs760518532	D	-	-	-	-	-
LOC100129697	2	+	+	-		16	89016677	G	C	Het.	Nonsynonymous SNV	c.G151C:p.V51L	rs71395347	D	-	-	-	-	-
LOC100129697	2	+	+	-		16	89017569	G	C	Het.	Nonsynonymous SNV	c.G1043C:p.G348A	rs34847212	D	-	-	-	-	-
NBPF10	2	+	+	+		1	145296478	G	T	Het.	Nonsynonymous SNV	c.G400T:p.D134Y	rs6663523	D	-	-	-	-	-
NPIPB11	2	+	+	+		16	29394758	G	T	Hom.	Nonsynonymous SNV	c.C1495A:p.P499T	-	D	-	-	-	-	-
PABPC3	2	+	+	-		13	25671271	AAGC	GAT	Het.	Frameshift substitution	c.935_938GAT	-	-	-	-	-	-	-
PDE4DIP	2	+	+	+		1	144852379	G	T	Het.	Nonsynonymous SNV	c.C7064A:p.P2355H	-	D	-	-	-	-	-
Family 3	No.	1	2	3															
	Status	A.	U.	U.															
CCDC7	2	+	+	+		10	33136820	AA	-	Het.	Frameshift deletion	c.1477_1478del:p.K493fs	-	-	-	-	-	-	-
CFAP46	2	+	+	+		10	134736204	G	A	Het.	Nonsynonymous SNV	c.C1265T:p.T422M	rs140185143	D	-	0.0022	-	-	-
CXorf23	2	+	-	+		X	19984574	G	T	Het.	Nonsynonymous SNV	c.C235A:p.P79T	rs143234295	-	D	0.0082	0.0299	-	-
LOC100129697	2	+	+	+		16	89017334	C	T	Het.	Nonsynonymous SNV	c.C808T:p.R270W	rs28617399	D	-	-	-	-	-
NBPF10	2	+	+	+		1	145296478	G	T	Het.	Nonsynonymous SNV	c.G400T:p.D134Y	rs6663523	D	-	-	-	-	-
NPIPB11	2	+	+	+		16	29394758	G	T	Hom.	Nonsynonymous SNV	c.C1495A:p.P499T	-	D	-	-	-	-	-
PABPC3	2	+	+	+		13	25671271	AAGC	GAT	Het.	Frameshift substitution	c.935_938GAT	-	-	-	-	-	-	-
SMIM13	2	+	+	+		6	11134683	C	T	Het.	Nonsynonymous SNV	c.C124T:p.R42W	-	-	D	-	-	-	-
Family 4	No.	1	2	3															
	Status	A.	A.	U.															
C16orf62	2	+	+	-		16	19566981	C	G	Het.	Nonsynonymous SNV	c.C197G:p.A66G	rs564734737	D	-	0.0002	-	-	-
GAGE2A	2	+	+	+		X	49237474	A	T	Het.	Nonsynonymous SNV	c.A179T:p.D60V	rs782582454	D	-	-	-	-	-
NPIPB11	2	+	+	+		16	29394758	G	T	Hom.	Nonsynonymous SNV	c.C1495A:p.P499T	-	D	-	-	-	-	-
PHIP	2	+	+	+		6	79671522	C	A	Het.	Nonsynonymous SNV	c.G3541T:p.A1181S	rs147526156	-	D	0.0002	-	-	-
SLC15A5	2	+	+	-		12	16430451	A	T	Het.	Nonsynonymous SNV	c.T169A:p.F57I	rs79942763	D	-	0.0066	0.0227	-	-
ZNF750	2	+	+	-		17	80789339	T	C	Het.	Nonsynonymous SNV	c.A992G:p.Y331C	-	-	D	-	-	-	-
Family 5	No.	1	2	3	4														
	Status	A.	U.	A.	U.													-	
ATP10B	2	+	+	+	+	5	160016684	A	G	Het.	Nonsynonymous SNV	c.T3665C:p.I1222T	rs144497343	-	D	0.0094	0.0061	-	-
NPIPB11	2	+	+	+	+	16	29394758	G	T	Hom.	Nonsynonymous SNV	c.C1495A:p.P499T	-	D	-	-	-	-	-
PIGN	2	+	+	+	-	18	59828420	G	A	Het.	Nonsynonymous SNV	c.C167T:p.A56V	rs61755362	-	D	0.0044	-	-	-
PRR14L	2	+	-	+	+	22	32108801	G	T	Het.	Nonsynonymous SNV	c.C5024A:p.A1675E	rs750572033	-	D	-	-	-	-

*A.: affected; U.: unaffected; Chr.: Chromosome; Ref.: Reference genome hg19; Het.: Heterozygote; Hom.: homozygote; D: Damaging variant

The 26 damaging variants affected 23 genes. The variants-affected genes are distributed in various functional categories, including RNA binding (*NBPF10*, *PABPC3*), transcriptional regulation (*ZNF750*), extracellular matrix (*CHST15*), structural protein (*NPIPB11*, *GRIP1*, *CFAP46*), and signal transduction (*PDE4DIP*, *PHIP*). As the examples, copy number change in NBPF10 is associated with multiple developmental and neurogenetic diseases, PABPC3 is involved in regulation of mRNA stability and translation initiation, and NPIPB11 is involved in forming nuclear pore complex. None of these genes are involved in DNA damaging repair pathways, in which the predisposition genes are traditionally considered to be located. Several variant-affected genes affected a few pathways mostly involved in housekeeping function. Whether any of these variant-affected genes can be predisposition gene candidates remains to be determined (Table 4, S4 Table).

Two homozygous damaging variants were present in *CHST15* and *NPIPB11*. The variant rs746518074 in *CHST15* was present in two affected members in Family 1, and the novel variant in *NPIPB11* was present in both affected and unaffected members in families 2, 3, 4 and 5. *CHST15* is an extracellular matrix component [26], and *NPIPB11* has unknown function. The high frequency of the novel variant in *NPIPB11* suggests that this variant is likely to be a normal homozygous polymorphism in Egyptian population. Little evidence exists for the roles of *CHST15* and *NPIPB11* in genetic predisposition in familial breast cancer.

We also compared the variant-affected genes with the mutation data from The Cancer Genome Atlas (TCGA) study [27]. Although none of the 825 breast cancer cases were marked as familial breast cancer cases, 49 germline mutations in the classical predisposition genes of *ATM*, *BRCA1*, *BRCA2*, *BRIPi*, *CHEK2*, *NBN*, *PTEN*, *RAD51C* and *TP53* were identified in 47 of 507 blood samples paired with breast cancer cases. None of the same variants were present in the Egyptian families we analyzed.

## Discussion

Decades’ study has well concluded that genetic predisposition plays the major roles in the development of familial breast cancer. As demonstrated by the extensive *BRCA* study, identification of the predisposition is essential for early diagnosis and prevention of breast cancer as it allows frequent monitoring the carrier health for early sign of the disease, blocking the tumorigenesis process by using chemo-prevention including tamoxifen and poly (ADP-ribose) polymerase (PARP) inhibitors, and applying preventive surgery to remove cancer susceptible tissues. Due largely to the scientific and economic advantages, current knowledge of genetic predisposition are largely derived from the developed countries of European and North American populations. Increased data from recent studies in Latino, Africa, and Asia populations demonstrate that genetic predisposition for familial breast cancer can be ethnic-specific in reflecting human evolution and geographic differences [4–10]. Without the information from different ethnic populations, our understanding of genetic predisposition for familial breast cancer will remain incomplete; and relying on the existing information as the solely references is not adequate to identify the patients from other ethnicities.

The Egyptian population has many unique genetic features developed during its evolution history and specific geographic location across Asian and African continents. Our study selected Egypt breast cancer families as a model to test if the genetic predisposition in populations of this area is the same as, or similar to, or very different from existing data of other ethnic populations. Our study showed the absence of mutations in *BRCA1*, *BRCA2*, and other classical predisposition genes, and the presence of the damaging variants in genes not involved in DNA damage repair in Egyptian patient families. We consider that the genetic predisposition in Egyptian familial breast cancer can be substantially different from the ones currently known from other ethnic populations.

Our study analyzed only five disease families. It is known that many predispositions are rare in the disease population. A possibility exists that certain known predispositions in the classical genes could be present in Egyptian familial breast cancer population but not detected due to the size limitation. Other possibility could be that the predisposition is located in non-coding region of the genome, which cannot be detected by exome sequencing method.

## Conclusions

Our study provides proof-of-principal evidence for the presence of specific genetic predisposition for familial breast cancer in Egyptian patients, and supports a scale-up study to characterize substantial numbers of disease families from local population in order to determine the nature of Egypt-specific predispositions in this population.

## Supporting Information

S1 Table Variants in other predisposition genes in familial breast cancer. (XLSX)

S2 Table Variants in *RECQL4* and *RRMB2*. (XLSX)

S3 Table Egyptian-specific normal variants. (XLSX)

S4 Table Coding-change variants in Egyptian familial breast cancer. (XLSX)

S5 Table Coding change variants in Caucasian familial breast cancer. (XLSX)

## Abbreviations

BIC: Breast Cancer Information Core
BRCAx: familial breast cancer without *BRCA1* and *BRCA2* mutation
ClinVar: public archive of interpretations of clinically relevant variants
MAF: minor allele frequency
